# Whole-Genome Sequencing and Annotation of *Bacillus safensis* RIT372 and *Pseudomonas oryzihabitans* RIT370 from *Capsicum annuum* (Bird’s Eye Chili) and *Capsicum chinense* (Yellow Lantern Chili), Respectively

**DOI:** 10.1128/genomeA.00288-15

**Published:** 2015-04-16

**Authors:** Huan You Gan, Han Ming Gan, Michael A. Savka, Alexander J. Triassi, Matthew S. Wheatley, Kubra F. Naqvi, Taylor E. Foxhall, Michael J. Anauo, Mariah L. Baldwin, Russell N. Burkhardt, Isabelle G. O’Bryon, Lucas K. Dailey, Nurfatini Idayu Busairi, Robert C. Keith, Megat Hazmah Megat Mazhar Khair, Muhammad Zamir Mohd Rasul, Nur Aiman Mohd Rosdi, James R. Mountzouros, Aleigha C. Rhoads, Melissa A. Selochan, Timur B. Tautanov, Steven J. Polter, Kayla D. Marks, Alexander A. Caraballo, André O. Hudson

**Affiliations:** a Monash University Malaysia Genomics Facility, Monash University Malaysia, Bandar Sunway, Petaling Jaya, Selangor, Malaysia; b School of Science, Monash University Malaysia, Bandar Sunway, Petaling Jaya, Selangor, Malaysia; c Thomas H. Gosnell School of Life Sciences, Rochester Institute of Technology, Rochester, New York, USA; d The School of Chemistry and Materials Science, Rochester Institute of Technology, Rochester, New York, USA; e The College of Health Science and Technology, Rochester Institute of Technology, Rochester, New York, USA

## Abstract

Here, we report the genome sequences of *Bacillus safensis* RIT372 and *Pseudomonas oryzihabitans* RIT370 from *Capsicum* spp. Annotation revealed gene clusters for the synthesis of bacilysin, lichensin, and bacillibactin and sporulation killing factor (*skfA*) in *Bacillus safensis* RIT372 and turnerbactin and carotenoid in *Pseudomonas oryzihabitans* RIT370.

## GENOME ANNOUNCEMENT

The plant genus *Capsium* (*Solanaceae*) is of interest given its use in a number of herbal remedies by indigenous inhabitants throughout the world and the ability to synthesize the antibacterial metabolite and the active ingredient capsaicin (8-methyl-*N*-vanillyl-6-nonenamide) which is predominantly stored in the fruit of the plant (1, 2). To gain some insights into bacteria that associate with the *Capsicum* spp., we embarked on a project as a laboratory exercise in the Fundamentals of Plant Biochemistry and Pathology course in the Biotechnology and Molecular Bioscience Program at the Rochester Institute of Technology to assess the bacterial diversity of bacteria associate with the fruits of *Capsicum annuum* (bird’s eye chili) and *Capsicum chinense* (yellow lantern chili). This resulted in the isolation and identification of *Bacillus safensis* RIT372 and *Pseudomonas oryzihabitans* RIT370 from surface sterilized internal tissue from the fruits that were purchased from a farmer’s market in Rochester, New York. The bacteria were initially identified as the *Bacillus* sp. and *Pseudomonas* sp. through the amplification and nucleotide sequence analysis of the variable 3 (V3) region from the 16S rRNA gene using the primers V3-forward 5′-ACTCCTACGGGAGGCAGCAG-3′ and V3-reverse 5′-ATTACCGCGGCTGCTGG-3 (3).

Genomic DNA (gDNA) from RIT372 and RIT370 was isolated from the bacteria using the GenElute bacterial genomic kit (Sigma-Aldrich, St. Louis, MO). The gDNA was prepared for whole-genome sequencing using a Nextera XT library preparation kit (Illumina, San Diego, CA). Whole-genome sequencing was performed using the Illumina Miseq (250-bp paired-end reads). The reads were error-corrected and assembled *de novo* using Spades 2.5 (4). The contigs were submitted to the RAST server for annotation. Genome-based taxonomic assignment of RIT372 and RIT370 was performed using JSpecies with an average nucleotide identity (ANI) cutoff value of >95% for species delineation (5). For RIT372, the *de novo* assembly and RAST annotation resulted in a genome size of 3,676,140 bp (38× coverage, 41.73% G+C content, *N*_50_ 70,686 bp, and 93 contigs), 3,787 open reading frames (ORFs), and 82 RNAs and for RIT370, 5,092,972 bp (30× coverage, 66.00% G+C content, *N*_50_ of 222,589 bp, 47 contigs), 66 RNAs, and 4,600 ORFs.

Secondary metabolite analysis of RIT372 and RIT370 using the antibiotics and secondary metabolite analysis shell (antiSMASH) tool resulted in the identification of gene clusters for the synthesis of bacilysin, where 60% of genes show homology in contig 2 between nucleotides 110,328 and 151,758, lichenysin, where 92% of genes show homology in contig 10 between nucleotides 8,808 and 92,529, bacillibactin, where 53% of genes show homology in contig 13 between nucleotides 10,936 and 60,646, sporulation killing factor, where 71% of genes show homology in contig 17 between nucleotides 59,844 and 77,860 for RIT372 and gene clusters for the synthesis of the siderophore turnerbactin, where 60% of genes show homology in contig 1 between nucleotides 158,226 and 211,070, and the terpene carotenoid, where 100% of genes show homology in contig 6 between nucleotides 41,781 and 65,405 for RIT370 (6, 7).

### Nucleotide sequence accession numbers. 

The whole-genome nucleotide sequences have been deposited at DDBJ/EMBL/GenBank under the accession numbers JYKW00000000 for *Bacillus safensis* RIT372 and JYKV00000000 for *Pseudomonas oryzihabitans* RIT370.
